# Measurement of Scattering and Absorption Cross Sections of Microspheres for Wavelengths between 240 nm and 800 nm

**DOI:** 10.6028/jres.118.001

**Published:** 2013-01-10

**Authors:** AK Gaigalas, Lili Wang, Steven Choquette

**Affiliations:** National Institute of Standards and Technology, Gaithersburg, MD 20899

**Keywords:** integrating sphere detector, Lorenz-Mie, microspheres, scattering

## Abstract

A commercial spectrometer with a 150 mm integrating sphere (IS) detector was used to estimate the scattering and absorption cross sections of monodisperse polystyrene microspheres suspended in water. Absorbance measurements were performed with the sample placed inside the IS detector. The styrene absorption was non zero for wavelengths less than 300 nm. Correction for fluorescence emission by styrene was carried out and the imaginary part of the index of refraction, n_i_, was obtained. Absorbance measurements with the sample placed outside the IS detector were sensitive to the loss of photons from the incident beam due to scattering. The absorbance data was fitted with Lorenz-Mie scattering cross section and a correction for the finite acceptance aperture of the spectrometer. The fit parameters were the diameter, the suspension concentration, and the real part of the index of refraction. The real part of the index was parameterized using an expansion in terms of powers of the inverse wavelength. The fits were excellent from 300 nm to 800 nm. By including the imaginary part obtained from the absorbance measurements below 300 nm, it was possible to obtain a good fit to the observed absorbance data over the region 240 nm to 800 nm. The value of n_i_ at 266 nm was about 0.0060±0.0016 for microspheres with diameters of 1.5 μm, 2.0 μm, and 3.0 μm. The scattering cross section, absorption cross section, and the quantum yield at 266 nm of microsphere with a diameter of 2.0 μm was 5.65±0.01 μm^2^, 1.54±0.03 μm^2^, and 0.027±0.002 respectively. The styrene absorption reduces the scattering cross section by 20 % at 266 nm.

## 1. Introduction

**Fig. 1:**
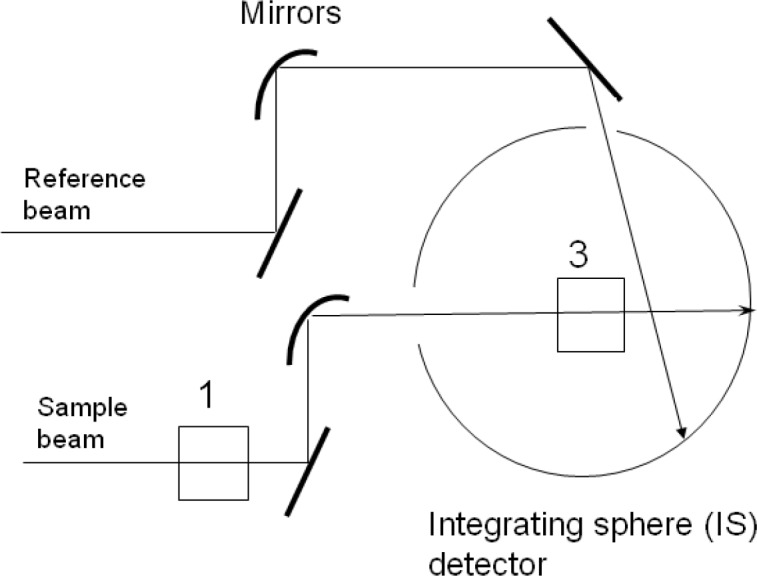
A schematic of the Perkin Elmer dual beam Lambda 850 spectrophotometer. The rectangle next to the number 1 represents the normal cuvette holder outside the integrating sphere (IS) detector. The rectangle next to the number 3 represents the cuvette holder inside the IS detector. For both cuvette positions, the same reference beam enters the IS detector through a reference port and hits the wall of the IS detector. In practice, the same ‘auto zero’ spectrometer function is used for both cuvette holders. The reference beam also has a cuvette holder which is not shown in the diagram. In addition, there is a cuvette holder in front of the IS sample beam entrance aperture which is also not shown.

In a previous paper [1] referred to as paper #1, it was demonstrated that the Lorenz-Mie (L-M) cross section for light scattering from monodisperse microspheres suspended in water gave a good description of absorbance measurements with a commercial spectrometer with an integrating sphere (IS) detector for wavelengths greater than 300 nm. The spectrometer configuration, shown in Fig. 1, allowed measurements with the suspension located outside the IS (holder 1 in Fig. 1) and measurements with the suspension located inside the IS (holder 3 in Fig. 1). In addition to the L-M cross section it was also necessary to include a description of the collection efficiency of forward scattered light which entered the IS detector and influenced the measurement of absorbance. With the suspension placed in the holder outside the IS, mainly transmitted light was collected by the IS detector, while in the case of the suspension placed inside the IS all of the light (transmitted and scattered) from the suspension was collected by the IS detector. In this paper we extend the analysis of the absorbance measurements to wavelengths below 300 nm and include the strong absorption band of styrene at 270 nm. Central to the analysis is the observation that when the cuvette containing the polystyrene (PS) microsphere suspension was placed inside the IS detector (holder 3), the entire measured absorbance was due to molecular absorption which was non-zero only for wavelengths below 300 nm. It was assumed that after a correction for fluorescence, the measured absorbance with the suspension placed inside the IS detector was equal to the molecular absorption. For wavelengths greater than 300 nm, the measured absorbance for the suspension placed inside the IS detector was within instrument uncertainty. In the present work, the absorbance measured in holder outside the IS in the wavelength range 240 nm to 300 nm was analyzed by including both absorption and scattering contributions. The analysis was divided into three parts. First, the analysis of the absorbance measured in the holder outside the IS above 300 nm was used to obtain the microsphere diameter, the real part of the index of refraction, and the concentration of the microsphere suspension. Second, the microsphere properties obtained for wavelengths above 300 nm were used for wavelengths below 300 nm to analyze the absorbance observed in the holder inside the IS. This latter analysis yielded an estimate of the imaginary part of the index of refraction. Finally, the results of the two steps were combined to analyze the absorbance measured in the holder outside the IS in the wavelength range 240 nm to 300 nm. In the following we describe a method to combine the measurements of absorbance for suspensions placed inside and outside the IS detector to obtain the complex index of refraction, the microsphere diameter, and the suspension concentration.

## 2. Interpretation of Integrating Sphere (IS) Measurements

The proposed method for measuring the scattering cross section of microspheres utilized a commercial spectrophotometer with an integrating sphere (IS) detector. The method was described previously [1] and here we provide only a short summary. The path of the light beam that probed the sample contained two mechanical cuvette holders labeled 1 and 3 in Fig. 1. The cuvette holder 1 was located outside of the IS detector in the normal sample compartment while the cuvette holder 3 was located inside the IS detector. The mirrors shaped the light beam so that it passes unobstructed through the cuvette holders. Measurements of absorbance were performed with the cuvette placed outside the IS (holder 1), and with the cuvette inside the IS (holder 3). The response in holder 3 was complicated by the detection of fluorescence, and possible biases due to settling of suspensions in holder 3 (no stirring).

The total extinction coefficient, *a=σN*, was expressed as a product of the total extinction cross section, *σ*, in units of cm^2^ and the concentration of microspheres, *N*, in units of cm^−3^. The path length through the cuvette was 1 cm. The total extinction coefficient was written as *a=a_s_* + *a_m_* where *a_s_* =*σ_s_N* is the apparent absorption coefficient due to scattering, and *a_m_=σ_m_N* is the molecular absorption coefficient. This description assumed that scattered photons were not detected and therefore appeared to have been absorbed. In most commercial spectrometers, the detector has a finite acceptance aperture and some of the forward scattered photons are detected. In order to correct for the detection of the forward scattered photons, the total extinction coefficient was modified as *a=a_s_* + *a_m_* − *a_sp_* with the definition *a_sp_=σ_sp_N* where *σ_sp_* is the integral of the differential scattering cross section over the angles subtended by the detector aperture at the location of the cuvette. In many cases, the measured total absorbance was less than 0.2 and the analysis could be simplified greatly by using a first order approximation 10^−*x*^ = 1 −*x*ln(10), which is valid for x<<1, and obtain the following approximate relation shown in Eq. (1).

(1) $$2.303\left(A-A_{\mathrm{buf}}\right)\approx a_{s}+a_{m}-a_{\mathrm{sp}}$$

Equation (1) was used to relate the measured absorbance (A) in any of the holders. (It is assumed that the buffer absorbance, *A_buf_*, was also measured in each holder) to the suspension properties embodied in the total extinction coefficient. To a good approximation, *a_sp_* ≈ 0 for the sample placed outside the IS, and *a_sp_* ≈ *a_s_* for the sample placed inside the IS. In order to compare the calculated cross section with the data, the values of the cross sections were multiplied by a concentration, a unit conversion factor 0.01, and 1/2.303 to obtain the expected absorbance. Assuming the validity of Eq. (1) and a cuvette path length of 1 cm, the relation between the measured absorbance and the calculated cross section is given by Eq. (2).

(2) $$\begin{array}{c} 2.303\left(A-A_{\mathrm{buf}}\right)=N\cdot 10^{6}cm^{-3}\cdot \sigma \cdot 10^{-12}m^{2}\cdot 10^{4}\frac{cm^{2}}{m^{2}}\cdot 1cm\\ =N\cdot \sigma \cdot 0.01 \end{array}$$

As an example, if measurements were performed on a cuvette with a suspension of microspheres with a concentration of 2.0 10^6^ cm^−3^, the calculated cross section was multiplied by a concentration 2.0 and 0.01/2.303 to give a predicted absorbance of about 0.13. In the following, the calculated cross sections will be presented in units of μm^2^, and the microsphere concentrations in units of 10^6^ cm^−3^. In the following section, Eq. (1) is used to analyze the measured extinction in holder 3 with *σ_s_* − *σ_sp_* set to zero.

## 3. Analysis of Absorbance Measurements from 240 nm to 800 nm

Measurements of absorbance of samples placed inside the IS showed that absorption occurred only for wavelengths below 300 nm. Therefore for wavelengths greater than 300 nm the measured absorbance for samples placed outside the IS was due to scattering and the analysis was performed using the L-M formalism with a real index of refraction. The fit to the data taken for samples outside the IS above 300 nm provided estimates of the microsphere diameter, real part of the index of refraction, suspension concentration, and the instrument acceptance angle. The next step involved the analysis of the absorbance measurement for samples placed inside the IS, which had only a contribution from molecular absorption. After correcting for fluorescence and residual background, the data acquired inside the IS was analyzed using the absorption cross section calculated according to the L-M formalism. The imaginary part of the index of refraction (below 300 nm) was assumed to possess a shape given by the function in Eq. (3).

(3) $$f\left(x\right)=\frac{1}{1+\;\text{exp}\left(350\left(x-\;0.272\right)\right)\;}\cdot \frac{1}{1+\;\text{exp}\left(-100\left(x-\;0.235\right)\right)}$$

The variable x stands for wavelength in units of μm to be consistent with the units used in the calculation of the L-M cross section. The function *f*(*x*) decreases for wavelengths greater than 272 nm and wavelengths less than 235 nm. In between these wavelengths the function has a plateau. The shape of the function reproduced the shape of the styrene absorption band centered on 270 nm and only a scale factor was needed to fit the absorbance taken in the holder inside the IS. The product of the scale factor and the function *f*(*x*) gave the imaginary part of the index of refraction needed to calculate the scattering cross section between 240 nm and 800 nm. The three main steps of the analysis are summarized below.

1. The data taken for the sample outside the IS in the region 300 nm to 800 nm was fitted to the L-M scattering cross section with the parameters: microsphere diameter, real part of the index of refraction, microsphere concentration, and the instrument acceptance angle.
2. The data taken for the sample inside the IS in the region 240 nm to 320 nm was fitted to the L-M absorption cross section using Eq. (3) to model the imaginary part of the index of refraction. The values of microsphere diameter, the real part of the index of refraction, and concentration determined in step 1 were used in this analysis.
3. The data taken for the sample outside the IS in the region 240 nm to 800 nm was compared to the calculated L-M scattering cross section with the parameters obtained in steps 1 and 2. The parameters include the microsphere diameter, real part of the index of refraction, imaginary part of the index of refraction, the microsphere concentration, and the instrument acceptance angle. There was no fitting in this step.

In what follows, the three steps of the analysis described above are presented in greater detail. Measurements were performed on PS microspheres suspended in aqueous PBS buffer or deionized water (DI). The suspensions were sufficiently dilute so that the absorbance was less than 0.2, and the simplified analysis given by Eq. (1) and Eq. (2) was used to model the data.

### 3.1 Analysis of the Absorbance Due to Scattering

**Fig. 2:**
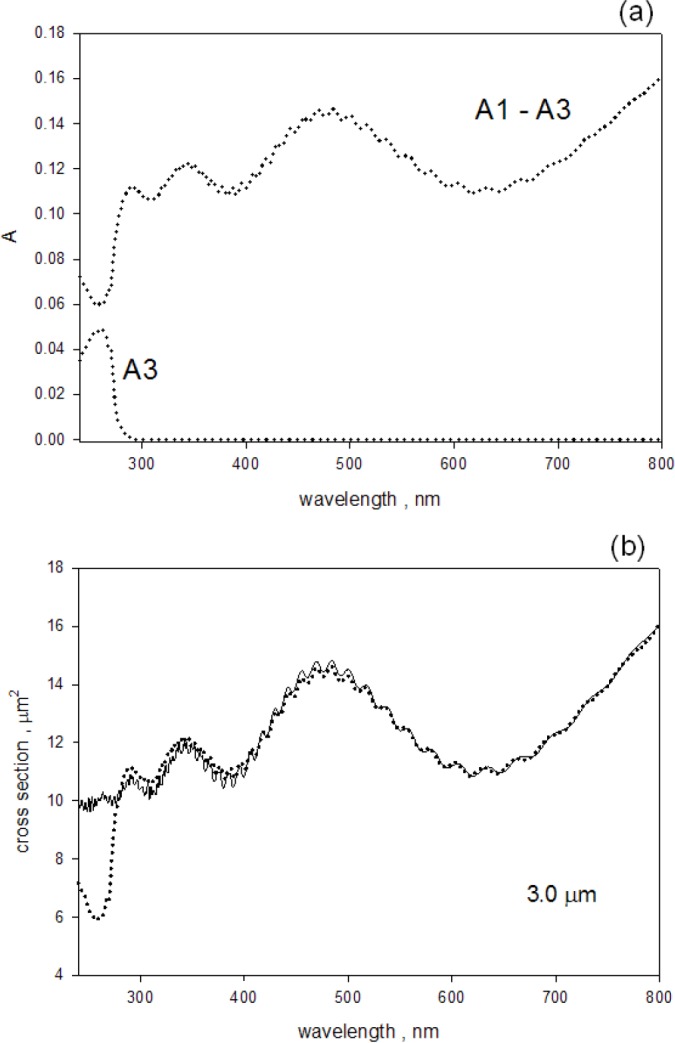
(a) The dotted trace labeled *A*_1_–*A*_3_ shows the measured absorbance due to scattering. The trace was obtained by subtracting the measured absorbance, *A*_3_, for a sample inside the IS from the measured absorbance, *A*_1_, for a sample outside the IS. In both cases, the buffer contribution was subtracted. The dotted trace labeled *A*_3_ shows the absorption due to styrene polymer. (b) The solid circles reproduce the trace labeled *A*_1_–*A*_3_ from part (a), and the solid trace is a best fit to the apparent L-M cross section for PS microspheres with a diameter of 3.0 μm.

Figure 2 shows measurements and analysis for a suspension of 3.0 μm microspheres in distilled water. The suspension was prepared by pipetting 10 μL of stock suspension obtained from Polysciences, Inc, (catalog number 17134, lot 621930) into 10 mL of distilled water^1^. The Lambda 850 spectrometer was scanned from 800 nm to 220 nm in increments of 1 nm with an integration time of 0.8 s, and a slit width of 2 nm. Four measurements were taken with a water sample and a suspension sample placed sequentially in holders outside and inside the IS. The solid circles in Fig. 2a show the measured absorbance due to scattering (trace A_1_–A_3_) and the absorbance due to molecular absorption (trace A_3_).The absorbance due to scattering was obtained by subtracting the measured absorbance of a sample placed inside the IS (A_3_) from the measured absorbance of the sample placed outside the IS (A_1_). In both cases, the measured absorbance of the cuvette filled with water was subtracted. The solid trace in Fig. 2b shows the best fit to the trace A_1_–A_3_ of a model based on the calculation of the L-M scattering cross section in the wavelength range from 300 nm to 800 nm. The Lorenz-Mie calculations were performed using MatLab with Maetzler [2] code for Mie scattering. The fit resulted in a diameter of 3.011 μm which was consistent with the value, (3.004±0.065) μm, provided by the manufacturer. The best fit shown in Fig. 2b was obtained from the minimization of residuals defined in Eq. (4).

(4) $$\begin{array}{l} Residuals=\sum_{\lambda }\left(\frac{A_{1}-A_{3}}{c}-Mie(d,n)+\int_{0}^{\Delta }\frac{d\sigma }{d\theta }(d,n,\theta )d\theta \right)\\ \;c=N\cdot 0.01/2.303\\ \;d=diameter\\ \;n=index\;of\;refraction\\ \;\Delta =acceptance\;angle \end{array}$$

The quantities *A*_1_ and *A*_3_ stand for the measured absorbance outside and inside the IS detector respectively (with the buffer contribution subtracted). The quantity *Mie(d,n)* is the calculated total scattering cross section. The integral of the differential L-M scattering cross section was performed over the angle subtended by the instrument entrance aperture at the location of the cuvette (symbol Δ in Eq. (4)). The integral models the effect of the finite acceptance aperture of the instrument. Because of the finite aperture some of the radiation scattered in the forward direction enters the instrument and reduces the measured absorbance. In what follows, the two calculated terms in Eq. (4) will be referred to as the apparent scattering cross section. The residuals in Eq. (4) were summed over a selected range of wavelengths with the lower bound set to a wavelength of about 300 nm. It was possible to obtain an excellent fit for wavelength region between 500 nm and 800 nm with a constant value of the index of refraction. For fitting below 500 nm, it was necessary to introduce a wavelength dependent index of refraction as described in paper #1. The fitting provided estimates of the diameter, real part of the index of refraction, concentration, and the acceptance angle. The imaginary part was set to zero for wavelengths greater than 300 nm. In order to obtain a good fit below 300 nm it was necessary to introduce an imaginary component to the index of refraction. The value of the imaginary component was obtained from analysis of *A*_3_ as described in the next section.

### 3.2 Measurement of Molecular Absorption in Scattering Suspensions

The comparison of absorbance measurements of samples placed inside and outside the IS detector showed that the measurements inside the IS detector were not sensitive to scattering losses. There was a small background absorbance (of the order of 0.003) which was most likely due to the escape of backward scattered photons through the entrance aperture of the IS detector. The background observed for measurements inside the IS was relatively constant over the wavelength region 300 nm to 800 nm, and we assumed that the background below 300 nm could be estimated from the scattering background spectrum between 300 nm to 800 nm. The trace *A*_3_ in Fig. 2a shows the result of measurements of absorbance inside the IS on a suspension of 3.0 μm microspheres. The trace labeled *A*_3_ in Fig. 2a was obtained by subtracting the buffer absorbance and an additional absorbance of 0.003 from the measured absorbance for the sample inside the IS. The absorbance value of 0.003 is close to the uncertainty introduced by variability of cuvette position and instrument drifts. Following the buffer and background subtractions, the *A*_3_ trace in Fig. 2a may still not represent the true absorbance because the emitted fluorescence from microspheres will reduce the observed absorbance.

#### 3.2.1 Fluorescence Correction of Absorbance Measured Inside the IS

**Fig. 3:**
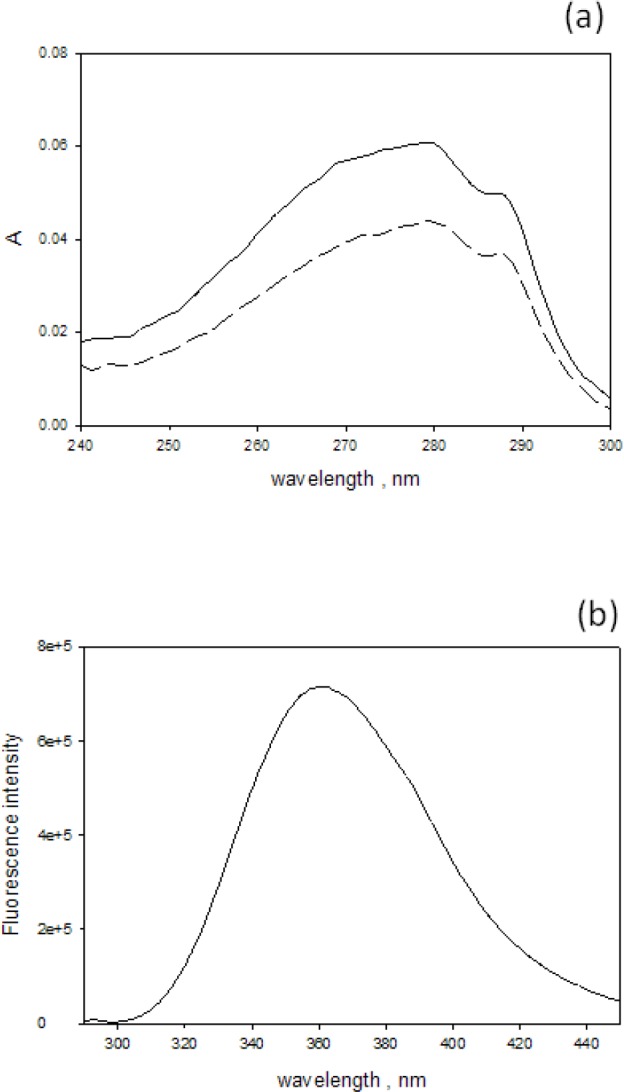
(a) The solid and dashed traces show the measured absorbance for 10 μmol/L aqueous tryptophan solution placed in holders outside (solid), and inside (dashed) the IS detector respectively. In both cases the buffer contribution was subtracted. There is a large difference in the measured absorbance in the two holders. The difference is due to fluorescence emission which is detected whenever the sample is inside the IS detector. The IS detector does not differentiate between the transmitted photons and the fluorescence photons thus recording a smaller absorbance for fluorescing samples. (b) The emitted fluorescence spectrum from a 10 μM tryptophan solution placed in a fluorimeter and excited with 266 nm light. The maximum emission is at 360 nm. The emitted fluorescence from either the microspheres or the tryptophan will reduce the recorded absorbance for samples place inside the IS detector.

**Fig. 4:**
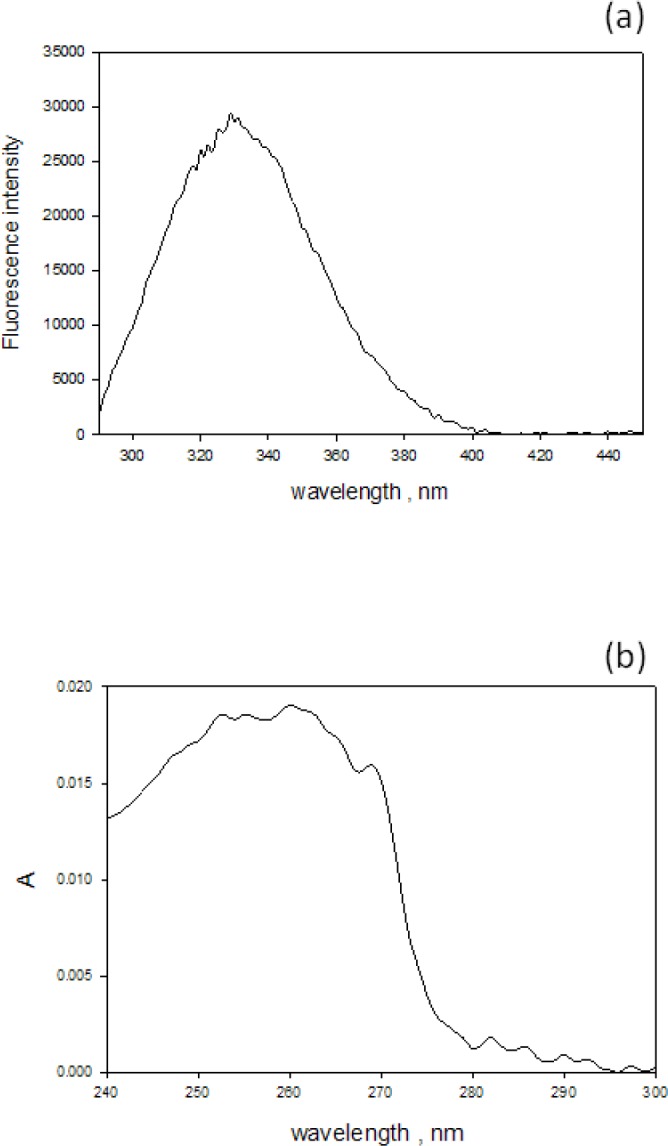
(a) The emitted fluorescence spectrum from a suspension of 1.5 μm microspheres placed in a fluorimeter and excited with 266 nm light. The maximum emission is at 350 nm. (b) The measured absorbance from a suspension of polystyrene microspheres with diameter 1.5 μm. The trace was obtained by placing the sample in the holder inside the IS detector and subtracting the buffer contribution and a small background contribution (0.003).

The effect of fluorescence on absorbance measurements for samples inside the IS is demonstrated in Fig. 3a. The solid and dashed traces in Fig. 3a show the absorbance measurements carried out for 10 mol/L aqueous solution of tryptophan placed outside and inside the IS respectively (the solution did not have measurable scattering). The reduction of the tryptophan absorbance measured inside the IS was attributed to the fluorescence emitted by the excited tryptophan molecules. Figure 3b shows the observed fluorescence emission when the same tryptophan solution was placed in a fluorimeter and excited with 266 nm light. Clearly the emitted tryptophan fluorescence will reduce the observed tryptophan absorbance measured inside the IS because the IS detector will not distinguish between photons transmitted through the cuvette and photons emitted by the solution inside the IS. Figure 4a shows the emitted fluorescence spectrum from a suspension of 1.5 μm microspheres placed in a fluorimeter and excited with 266 nm light. A comparison of the spectra in Fig. 4a and Fig. 3b shows that the fluorescence emission from the microspheres is similar to the fluorescence emission from the tryptophan solution. Therefore it was assumed that the measure absorbance of a suspension of 1.5 μm microspheres shown in Fig. 4b would include a reduction by the emitted fluorescence and that the actual absorbance of the microsphere suspension is larger. In order to quantify the reduction in the measured absorbance due to fluorescence, it was assumed that the relative reduction in absorbance is proportionate to the quantum yield as indicated in Eq. (5).

(5) $$\frac{A-A_{3}}{A}=m\Phi$$

Here *A* is the true absorbance, *A*_3_ is the absorbance measured inside the IS, Φ is the quantum yield, and *m* is the proportionality constant. In the ideal case where every photon is detected with the same probability, the value of *m* should be 1 since if the quantum yield is 1, every absorption leads to an emitted photon and the measured absorbance, *A*_3_, would be 0. In practice, the photon detection probability varies significantly in the UV region so that the value of *m* will be different from 1 as discussed in a previous work [3]. Equation (5) was treated as a phenomenological relation. The tryptophan measurements shown in Fig. 3a were used to estimate the proportionality constant *m* because the true absorption of tryptophan could be obtained from measurements in holder outside the IS while the quantum yield of tryptophan has been determined to be 0.13 [4,5]. Explicitly, the proportionality constant was assumed to be given by Eq. (6).

(6) $$\frac{A_{1}-A_{3}}{A_{1}}=m\cdot 0.13$$

**Fig. 5:**
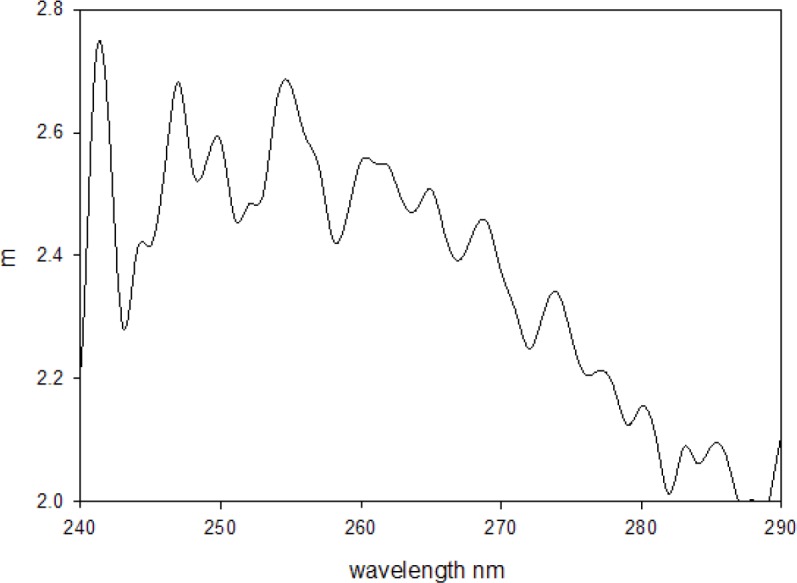
The data shown in Fig. 3a was used to form the ratio, *m*, given by Eq. (6) in the text. The quantity 0.13 in Eq. (6) is the quantum yield of tryptophan in aqueous solution. The ratio, *m*, characterizes the effect of fluorescence emission on the observed absorbance of tryptophan samples placed inside the IS detector. Ideally the ratio should be constant. The variation with wavelength reflects the difference in spectral response between the fluorescence and the incident light.

Where *A*_1_ and *A*_3_ are the absorbance measurements obtained outside and inside the IS as shown in Fig. 3a. Figure 5 shows the result of applying Eq. (6) to the data in Fig. 3a. From the trace in Fig. 5 it is clear that the quantity *m* in Eq. (6) is not a constant and varies from 2.5 to 2.0 over the tryptophan absorption band. We will use *m* = 2.4 in the estimate of the true absorbance of microspheres. This choice is motivated by the desire to minimize systematic errors in the vicinity of 266 nm. It was also assumed that the emission spectra of tryptophan and the microspheres were sufficiently similar so that spectral response corrections could be ignored in the first approximation. The quantum yield of microspheres was obtained relative to the quantum yield of tryptophan by using the relation given in Eq. (7) [6].

(7) $$\frac{I}{I_{\mathrm{ref}}}=\frac{A}{A_{\mathrm{ref}}}\frac{\Phi }{\Phi_{\mathrm{ref}}}\frac{n^{2}}{n^{2}{}_{\mathrm{ref}}}$$

Here *I* and *I_ref_* are the integrated fluorescence intensities obtained by summing the spectra shown in Fig. 4a (*I*) and 3b (*I_ref_*). *A* and *A_ref_* are the absorbance values at 266 nm of the microsphere suspension and tryptophan reference solution respectively. The indexes of refraction *n* and *n_ref_* refer to the material in the sample cuvette and are both very close to the value for water, hence their ratio was set to 1 in Eq. (7). Equation (7) indicates that the value of *A* is needed to find the microsphere quantum yield Φ, while Eq. (5) states that Φ is needed to determine the true microsphere absorption *A*. Solving Eq. (7) for Φ in terms of *A*, and substituting into Eq. (5) lead to an estimate of the true absorbance of the microsphere suspension given by Eq. (8).

(8) $$A=A_{3}+m\frac{I}{I_{\mathrm{ref}}}A_{\mathrm{ref}}\Phi_{\mathrm{ref}}$$

Inserting the various measured values into Eq. (8) resulted in an increase in *A*_3_ of only several percent. Inserting the corrected absorbance of microspheres into Eq. (7) lead to a quantum yield of about 0.018 for microspheres with diameter of 1.5 μm. Repeated measurements for microspheres with a diameter of 2.0 μm gave a quantum yield of 0.026±0.004. If the uncertainty in the measurements for the 1.5 μm microspheres is assumed to be ±0.004 then the two measurements are consistent. Alternately it may be that the QY for the smaller microspheres is smaller due to the decrease in the density of final states. The quantum yield of styrene monomers in dilute solution is 0.24 [7]. However as the molecular weight of the styrene polymers increase, the quantum yield decreases, reaching a limit of 0.018 for very long strands [8]. Therefore there is a substantial quenching of fluorescence in the styrene polymer and the observed quantum yield for PS microspheres is reasonable. Since the correction of absorbance measurement due to fluorescence emission is so small, the values of *A*_3_ in Fig. 4b were used to represent the “true” absorbance of the microsphere suspension. A second correction that was neglected was the increase in the measured absorbance *A*_3_ due to the repeated passage of the light reflected inside the IS through the cuvette inside the IS. This correction was expected to be small because the suspension has a small absorption value (Fig. 1a). This was collaborated by measurements of absorbing, non-fluorescing solutions (e.g. myoglobin or fluorescein in pH 5 buffer) which gave indistinguishable responses outside and inside the IS detector [3].

#### 3.2.2 Analysis of the Microsphere Absorbance Measurements

The microsphere absorbance recorded inside the IS (an example shown in Fig. 4b) was assumed to be the true molecular absorption and it was used to obtain the microsphere absorption cross section using the relation shown in Eq. (9).

(9) $$\begin{array}{l} Residuals=\sum_{\lambda }{\left(\frac{A_{3}-A_{\mathrm{buf}}}{c}-Mie(d,n)\right)}^{2}\\ \;c=N\cdot 0.01/2.303\\ \;d=diameter\\ \;n=index\;of\;refraction \end{array}$$

**Fig. 6:**
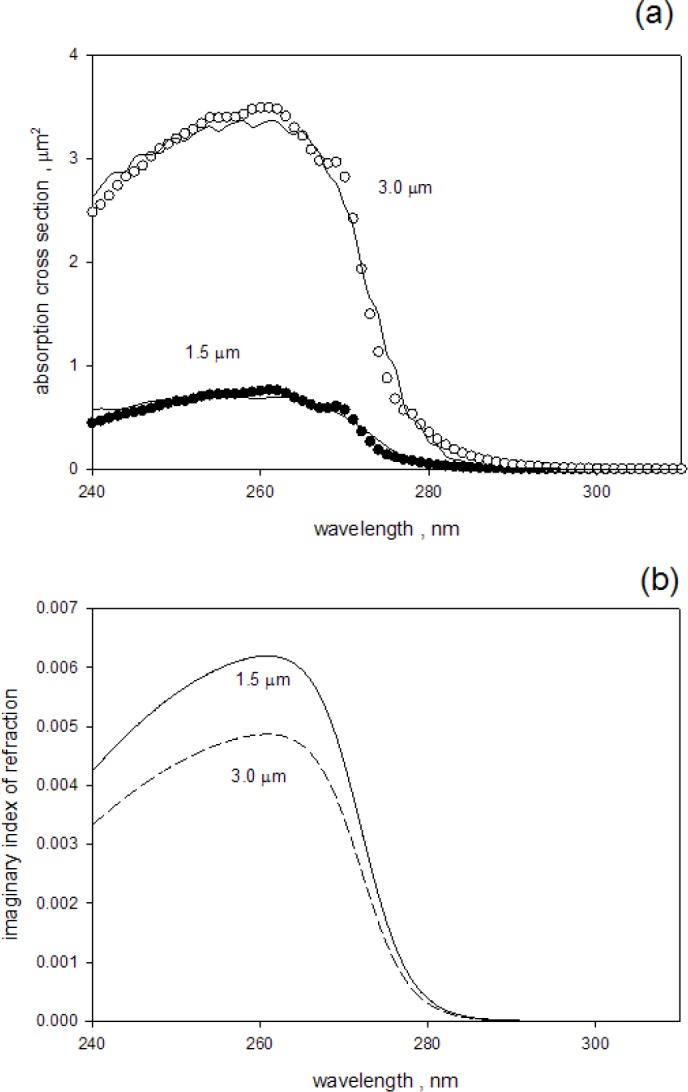
(a) The solid and open circles show the estimated absorption cross sections of suspensions of 1.5 μm and 3.0 μm microspheres respectively. The estimates were obtained by dividing the measured absorbance of the samples inside the IS by parameter *c* in Eq. (9). The parameter *c* is proportionate to the microsphere concentration. The solid traces show the absorption cross section which best fits the data. The fitting was performed according to Eq. (9) in the text. (b) The imaginary component of the index of refraction for 1.5 μm (solid trace) and 3.0 μm (dashed trace) microspheres obtained from the best fit to the data shown in part (a).

Where *Mie(d,n)* calculates the total absorption cross section for a given set of parameters. The parameters *c, d*, and the real part of *n* were taken from the fits to the measured absorbance outside the IS for wavelengths greater than 300 nm. The functional shape of the imaginary component of the index of refraction was taken as function *f*(*x*) given in Eq. (3). The function *f*(*x*) was multiplied by a scaling parameter in order to fit the measured absorbance. The solid and open circles in Fig. 6a show the estimated absorption cross section for 1.5 μm and 3.0 μm microspheres respectively obtained by dividing the measured absorbance by the parameter *c* in Eq. (9). The solid traces in Fig. 6a show the best fits to the data using the imaginary index of refraction given by Eq. (3). Figure 6b shows the imaginary part of the index of refraction which best described the absorption cross section shown in Fig. 6a. The comparison of the absorption cross sections in Fig. 6a shows that for 266 nm the absorption cross section of the 3.0 μm microsphere is only four times larger than the absorption cross section of the 1.5 μm microsphere, whereas the volume is eight times larger. The greater absorption efficiency of the 1.5 μm microsphere accounts for the factor of two. The comparison of the imaginary components of the index of refraction in Fig. 6b shows that the values for the 1.5 μm microsphere are about 1.4 times larger than the values for the 3.0 μm microsphere. In principle the two values should be the same since the imaginary part of the index of refraction is a property of the polymer material which is presumably identical for the two microspheres. It is likely that the difference in the imaginary part of the index of refraction is due to systematic uncertainties inherent in the measurement and analysis. A central problem, which has been mentioned before, is the estimate of the efficiency of collection of forward scattered light by the detector with a finite aperture.

### 3.3 Estimate of the Scattering Cross Section between 240 nm and 800 nm

**Fig. 7:**
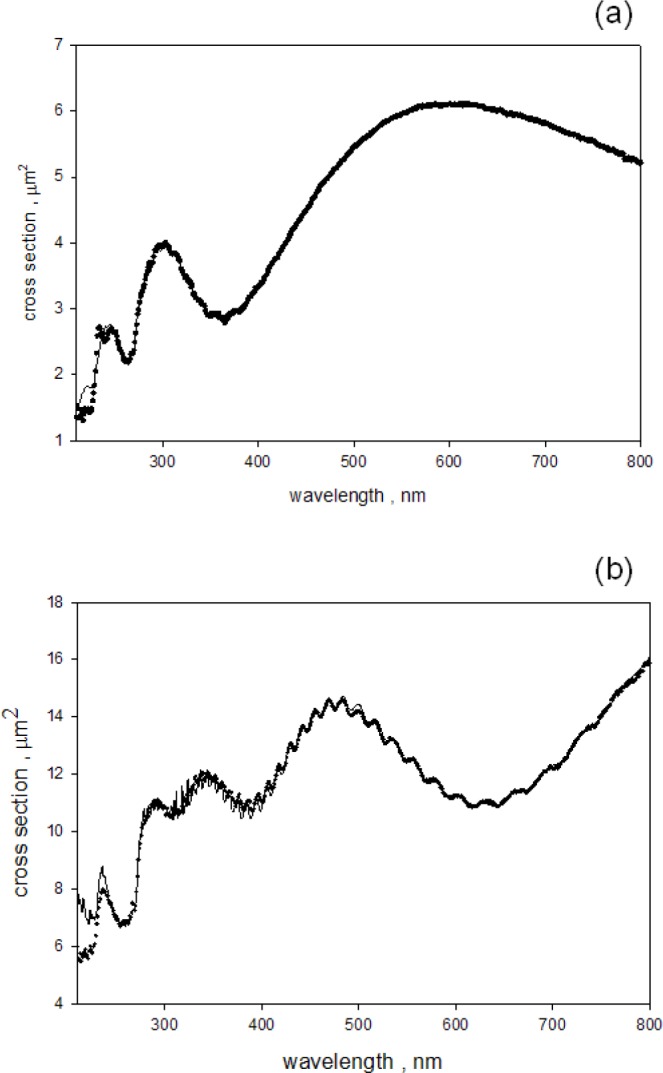
(a) The solid circles show the estimated apparent scattering cross section of a suspension of 1.5 μm microspheres placed outside the IS. The estimate was obtained by dividing the measured absorbance by the parameter *c* in Eq. (4) (the buffer contributions were subtracted). The solid trace shows the calculated apparent scattering cross section which best fits the data. The fitting was performed according to Eq. (4) in the text. For wavelengths less than 300 nm, the imaginary component of the index of refraction shown in Fig. 6b was used in the calculation of the M-L scattering cross section. The apparent scattering cross section at 266 nm is reduced from the value it would have if there was no molecular absorption. (b) The same comparison as in Fig. 7a except the diameter of the microspheres was 3.0 μm. Again, the apparent scattering cross section at 266 nm is reduced from the value it would have if there was no molecular absorption.

In the third and final part of the analysis, the fit to the scattering cross section above 300 nm and the fit to the absorption cross section below 300 nm were combined to obtain an estimate to the scattering cross section in the wavelength range 240 nm to 800 nm. There was no additional fitting, the parameters obtained for the fits above and below 300 nm were combined to obtain the response over the entire range. The solid circles in Fig. 7a show the scattering cross section obtained for 1.5 μm microspheres and the solid trace shows the best fit for wavelengths between 240 nm and 800 nm. Figure 7b gives the same information for microspheres with diameter of 3.0 μm. Comparison of Fig. 2b and Fig. 7b shows that including the imaginary component of the index of refraction leads to a much better correspondence between the measurements and the calculated response. The styrene absorption band at 270 nm produces a significant reduction in the scattering cross section at 270 nm. The reduction is expected because the scattering and absorption processes are mutually exclusive. The absorption of photons by the styrene molecules reduces the number of observed scattered photons. Conversely the observation of a scattered photon implies that absorption did not occur.

**Fig. 8:**
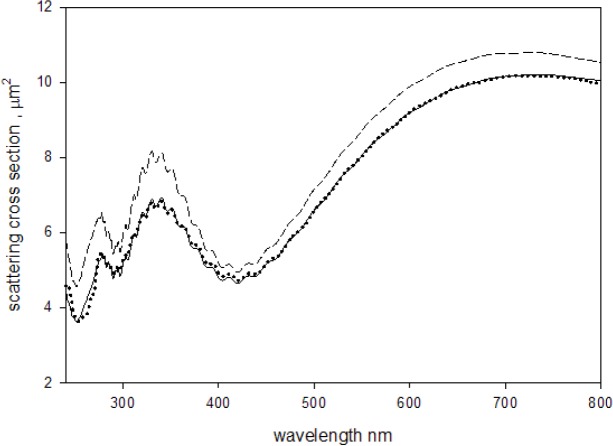
The solid circles show the measured absorbance (divided by the parameter *c* in Eq. (4)) of a suspension of 2.0 μm microspheres placed outside the IS. The solid trace shows the computed apparent scattering cross section according to Eq. (4). For wavelengths less than 300 nm, the imaginary component of the index of refraction was similar to that shown in Fig. 6b. The total scattering cross section (dashed trace) was calculated using the same parameters as in the calculation of the apparent scattering cross section (solid trace) except for the instrument acceptance angle which was set to zero. The total scattering cross section at 266 nm is reduced from the value it would have if there was no molecular absorption.

The three steps of the analysis culminated in the estimate of the properties of the microspheres and the instrument which yielded a good representation of the measured data. The solid circles in Fig. 8 show the measured absorbance for a suspension of 2.0 μm microspheres divided by the fit parameter *c*, and the solid trace shows the calculated apparent scattering cross section. The dashed trace in Fig. 8 shows the calculated L-M total scattering cross section for 2.0 μm polystyrene microspheres in water. The calculation of the total scattering cross section (dashed trace) used the same parameters as the calculation of the apparent cross section except for the instrument acceptance angle which was set to zero (this parameter characterizes the instrument and was not needed to calculate the total scattering cross section). Table 1 gives the results of four consecutive independent measurements performed on suspensions of 2.0 μm microspheres. The suspensions were made by pipetting 5 μL of stock suspension (Polysciences Cat. No. 19814, lot no. 625383) into 20 mL distilled water. The values of the cross sections in Table 1 were obtained by using the best fit parameters from Eq. (4) and Eq. (9) and then setting the acceptance angle to zero. The quantum yield (QY) was obtained with additional fluorescence measurements using Eq. (7) and Eq. (8). The real part of the index of refraction was obtained by inserting the fit parameters into Eq. (11) of paper #1.

The value of the diameter is consistent with the value (1.898±0.029) μm provided by the manufacturer of the microspheres. The reproducibility of the various properties is excellent as shown by the values of the standard deviation (SD) in the last row of Table 1. However the values of the index of refraction contain systematic uncertainties due to the uncertainty in the estimate of the collection efficiency of forward scattered light. This uncertainty leads to uncertainty in the *c* parameter which scales the measured absorbance to the scattering or absorption cross sections. The systematic uncertainty becomes apparent if a comparison is made between the imaginary component of the index of refraction shown in Table 1 and the average value of the imaginary part of the index of refraction, obtained from measurements on microspheres with diameters 1.5 μm, 2.0 μm, and 3.0 μm, which is 0.0060±0.0016. Thus the uncertainty due to systematic effects is substantially larger than the uncertainty due to random variation in independent measurements on microspheres with the same diameter.

Since the L-M formalism uses only the ratio of the index of refraction of the microsphere and the index of refraction of the medium, the calculation of the total cross section can be extended to microspheres in air by simply setting the medium index of refraction to 1 and retaining the values of the diameter and the microsphere index of refraction.

## 4. Conclusion

An analysis of the measurement process with a spectrometer with an integrating sphere (IS) detector lead to a procedure for separating the measured absorbance into a part due to scattering and a part due to molecular absorption. The analysis hinged on the interpretation of absorbance measured for a suspension placed outside (*A*_1_) and inside (*A*_3_) the IS detector of the spectrometer. Equation (1) and Eq. (2) give the relationship between the measured absorbencies (*A*_1_, *A*_3_) and the analyte properties (*a_s_*, *a_m_*, *a_sp_*). The results suggest that the measurement model presented in this work is valid and that it is indeed possible to separate the contributions from scattering, *a_s_*, and molecular absorption, *a_m_*. The two quantities, *a_s_*, *a_m_*, are independent characteristics of the microsphere suspension. The quantity *a_m_*, which is directly related to the absorption cross section, gives information about the electronic states of the absorbing styrene molecules, while the quantity *a_s_*, which is directly related to the scattering cross section, provides information about the diameter and the wavelength dependence of the index of refraction of the material inside the microsphere. Further work is needed to clarify the systematic errors inherent in the preparation of suspension samples, instrument response, and the measurement model. Most likely the most significant uncertainty is in the estimate of the efficiency of collection of forward scattered light, which is directly related to the partial scattering extinction *a_sp_*. The collection efficiency depends on the instrument configuration as well as the angular distribution of the scattered radiation. A better parameterization of the collection efficiency of forward scattered light will be addressed in future work.

## Figures and Tables

**Table 1:** Properties of 2.0 μm microspheres at 266 nm obtained from the fit of the L-M apparent cross section to the absorbance measurements

Trial	Diameter μm	*n* real	*n* imaginary	Scattering cross section, μm^2^	Absorption cross section, μm^2^	QY
1	1.943	1.761	7.726e-3	5.65	1.56	
2	1.942	1.762	7.730e-3	5.64	1.54	0.026
3	1.942	1.762	7.730e-3	5.64	1.54	0.027
4	1.943	1.761	7.726e-3	5.66	1.50	0.029

Mean	1.9426	1.7615	7.7280e-3	5.648	1.535	0.0273
SD	6.75e-4	5.77e-4	2.31e-6	9.57e-3	0.0252	1.53e-3

## Footnotes

1 Certain commercial equipment, instruments, and materials are identified in this paper to foster understanding. Such identification does not imply recommendation or endorsement by the National Institute of Standards and Technology.

## References

[1] Gaigalas AK (2012). Measurement of Scattering Cross Section with a Spectrometer with an Integrating Sphere Detector. J Res Natl Inst Stand Technol.

[2] Maetzler C (2002). MATLAB Functions for Mie Scattering and Absorption.

[3] Gaigalas AK, Wang L (2008). Measurement of the Fluorescence Quantum Yield Using a Spectrometer With an Integrating Sphere Detector. J Res Natl Inst Stand Technol.

[4] Kirby EP, Steiner RF (1970). The Influence of Solvent and Temperature upon the Fluorescence of Indole Derivative. The Journal of Physical Chemistry.

[5] Chen RF (1972). Measurement of Absolute Values in Biochemical Fluorescence Spectroscopy. J Res Natl Bur Stand.

[6] Ediger MD (1982). On the Refractive Index Correction in Luminescence Spectroscopy. Chemical Physics Letters.

[7] Condirston DA, Laposa JD (1979). Fluorescence Quantum Yields and Lifetimes of Styrene at 298 and 77 K. Chemical Physics Letters.

[8] Ishii T, Handa T, Matsunaga S (1978). Effect of Molecular Weight on Excimer Formation of Polystyrenes in Solution. Macromolecules.

